# Thalamic homeostatic transcriptomic signatures are altered in a mouse model of cholestatic liver injury and are mitigated by systemic TNF neutralization

**DOI:** 10.1186/s13041-026-01302-5

**Published:** 2026-04-18

**Authors:** Wagdi Almishri, Jeff F. Dunn, Mark G. Swain

**Affiliations:** 1 Department of Medicine, Snyder Institute for Chronic Diseases, Cumming School of Medicine, University of Calgary, Calgary, AB Canada; 2 Hotchkiss Brain Institute, Department of Radiology, Experimental Imaging Centre, Cumming School of Medicine, University of Calgary, Calgary, AB Canada; 3 Calgary Liver Unit, Department of Medicine, Snyder Institute for Chronic Diseases, Cumming School of Medicine, University of Calgary, Calgary, AB Canada

**Keywords:** Fatigue, Brain, Liver-brain axis, Sickness‑behavior, Immune-mediated liver diseases, Cholestasis

## Abstract

Cholestatic liver diseases (CLD), including PBC and PSC, are frequently associated with debilitating sickness‑behavior symptoms such as fatigue, cognitive impairment, and anxiety/depression, which have poorly defined etiology and limited treatment options, substantially reducing quality of life. Across immune‑mediated diseases, thalamic changes have been well documented and found to correlate with a number of theses symptoms. Changes in thalamic structure and neural connectivity have been previously identified in PBC patients by us and other groups. These changes include findings indicating reduced tissue neuronal density and myelination, decreased thalamic size, and changes in functional neural connectivity between the thalamus and basal ganglia and cortical behavior-regulating areas that correlated with symptom severity. These observations implicate altered thalamic structure and function in the genesis of CLD-related sickness‑behavior symptoms. Therefore, we used a well characterized mouse model of CLD due to bile duct ligation (BDL) to mechanistically examine how CLD impacts thalamic structure and function. BDL mice showed reduced thalamic volume compared to sham-ligated controls, as determined by MRI, and an altered thalamic RNA–seq transcriptomic signature with predicted molecular activity consistent with inhibition of cellular growth, proliferation, neurite formation, neural function, and myelination, as well as enhanced apoptosis. Additionally, BDL was associated with changes in gene expression for key thalamic nervous system signaling pathways that regulate neurotransmission and behavior. We have previously demonstrated that systemic TNF is a key regulator of liver-to-brain communication and the development of adverse behavioral symptoms in BDL mice. Therefore, we administered anti-TNF antibody to neutralize systemic TNF in BDL mice and determined the impact on thalamic transcriptomic changes. TNF neutralization attenuated BDL-associated thalamic transcriptomic changes and enhanced gene expression in pathways regulating neurotransmission, cell proliferation, and those associated with neuron survival, although myelination pathways remained unaltered. We show that reduced thalamic volume in BDL mice is associated with transcriptomic alterations suggesting inhibition of structural machinery and dysfunction of neural signaling; findings that are significantly attenuated after systemic TNF neutralization. Our findings suggest that TNF inhibition may represent a potential novel approach to attenuate thalamic changes in CLD.

**Supplementary Information:**

The online version contains supplementary material available at 10.1186/s13041-026-01302-5.

## Introduction

Primary biliary cholangitis (PBC) is a chronic liver disease marked by immune-mediated destruction of small intrahepatic bile ducts leading to cholestasis, progressive fibrosis, and potentially cirrhosis and liver failure [1]. In addition to liver injury, PBC patients commonly experience debilitating behavioral and neuropsychiatric symptoms, including fatigue, cognitive impairment, and anxiety/depression, that can profoundly impair quality of life [1]. Indeed, fatigue can affect up to 80% of PBC patients, is considered moderate to severe in roughly 40% [2, 3], and is characterized by extreme tiredness, decreased motivation, impaired cognition, and often social withdrawal [4, 5]. Fatigue disproportionately impacts younger women, is independent of liver disease severity or therapeutic response to ursodeoxycholic acid (UDCA) treatment [4, 5], and commonly persists post-liver transplant [6]. Unfortunately, how cognitive and behavioural symptoms develop in the context of immune‑mediated liver injury in patients with PBC remains poorly understood. However, thalamic dysfunction is increasingly implicated as an important mechanism regulating behavioural symptom development in the context of many chronic inflammatory diseases.

The thalamus is a small brain structure situated at the top of the brainstem, traditionally considered as the key relay station for nearly all sensory and motor signals in transit from the body to the cerebral cortex [7]. However, the thalamus is not simply a passive relay station but acts as a dynamic integrator and processor of neural information [8, 9], reciprocally sharing information between the cortex and subcortical structures, including the limbic system, striatum and basal ganglia [10, 11]. These neural interactions critically regulate numerous complex behaviors and have been strongly implicated in the genesis of central fatigue. Specifically, altered thalamic volume and neural connectivity have been linked to both subjective and objective measures of fatigue in immune-mediated inflammatory diseases, including long-Covid, and in neurological diseases such as multiple sclerosis [12–15]. Changes in thalamic structure and neural connectivity have been previously identified in PBC patients by us and other groups. Specifically, PBC patients showed increased thalamic apparent diffusion coefficient compared to healthy controls [16], a finding indicating reduced tissue neuronal density and decreased myelination [17]. Using resting state functional MRI we found decreased thalamic size and intrinsic neural activity in PBC patients associated with changes in functional neural connectivity between the thalamus and the striatum, limbic structures, and key behavior-regulating areas of the cortex that correlated with symptom severity, including fatigue [1, 18]. These observations implicate altered thalamic structure and function in the genesis of PBC-related fatigue.

How immune-mediated liver injury in PBC might drive changes in brain function is poorly understood. However, communication pathways between the liver and brain must be established in PBC that generate altered CNS neurotransmission leading to the development of central fatigue [19–21]. Indeed, we previously showed in an animal model of PBC due to bile duct ligation that blocking systemic TNF signaling significantly blunted altered brain neurotransmission and behavioral changes associated with cholestatic liver injury in this animal model, findings directly implicating TNF as an important liver-to-brain signaling molecule in regulating the CNS manifestations associated with cholestatic liver disease [22–25]. However, the potential role of TNF in regulating thalamic changes in PBC remains unknown but is clearly important as therapeutic targeting of TNF signaling is a feasible approach that could be potentially employed clinically.

A homeostatic thalamic transcriptomic signature maintains normal thalamic structure and function [26]. We hypothesize that cholestatic liver disease leads to changes in this homeostatic gene signature resulting in altered thalamic structure and neural function, similar to that identified in other chronic systemic immune-mediated diseases [12–15]. Moreover, changes we identify in the thalamic transcriptomic landscape could potentially be used as an experimental tool to define key systemic liver-to-brain signaling pathways, such as those involving TNF in driving these thalamic changes in cholestatic liver disease. Therefore, we undertook a series of experiments using the bile duct ligation mouse model of cholestatic liver disease to examine this.

## Methods and materials

### Animal model of cholestatic liver injury

Bile duct ligation (BDL) in rodents is a well-established experimental model of cholestatic liver injury characterized by progressive liver damage, ductular reaction, inflammation, fibrogenesis, and related systemic alterations [27–29]. In addition, BDL mice exhibit highly reproducible changes in brain neurotransmission and behavior that mimic a number of clinical manifestations widely documented in PBC patients [4, 5, 22–25, 30–33]. Therefore, in this study we used the BDL mouse model to define cholestatic liver injury-associated molecular and structural changes within the thalamus. Male C57BL/6 mice (8–10 weeks old; Jackson Laboratory, Bar Harbor, ME) underwent BDL and sham surgeries according to our previously established experimental protocols [22–25, 33]. Control mice underwent sham resection that included laparotomy with manipulation of the bile duct without ligation. All surgical procedures were performed under isoflurane anesthesia, and measures were taken to minimize animal suffering. All MRI, transcriptomic, and PCR analyses were conducted 10 days following surgery [23, 33]. Numbers of animals used in each experiment are specified in the corresponding figure legends. All experimental procedures were approved by the University of Calgary Animal Care Committee and conducted in accordance with the guidelines of the Canadian Council on Animal Care.

## In vivo brain magnetic resonance imaging (MRI)

In vivo MRI imaging was conducted in the Experimental Imaging Centre (EIC), University of Calgary, using a 9.4T/21 cm horizontal bore magnet (Magnex, UK) with Bruker B-GA12S gradient insert and Bruker Avance II Biospin MR imaging system run by the ParaVision 5.1 software. Bruker’s 20 mm 1 H Mouse Brain Quadrature Transmit/Receive Surface CryoProbe cooled by a closed-cycle refrigeration system, was used for imaging. Complete descriptions of the in vivo MRI acquisition parameters, procedural details, and image‑analysis workflow are provided in the Supplementary section.

## Thalamic transcriptome analysis

Mice were euthanized with isoflurane and perfused with 20 ml of ice-cold PBS. The whole brain was removed, and the thalamus was dissected and stored at -80 °C in RA1 lysis buffer (Cat No. 740961.500; Macherey–Nagel, Düren, Germany) until RNA extraction. Distinct cohorts of mice were used for the MRI study and RT-qPCR/RNA-Seq analyses. Total RNA was extracted from the thalamus using the NucleoSpin^®^ RNA purification kit (Cat No. 740955-250, Macherey–Nagel, Düren, Germany). Bulk tissue RNA sequencing on thalamus RNA was conducted in the University of Calgary Centre for Health Genomics and Informatics, as previously described [33]. RNA libraries were sequenced using paired-end 50 bp fragment sequencing on NovaSeq™ 6000 and NextSeq 2000 high-throughput Illumina sequencing systems.

Bulk RNA-seq analysis was performed using CLC Genomics Workbench version 24.0.2. Statistical comparisons of gene expression tracks generated by CLC Genomics Workbench were exported to the web-based Ingenuity Pathway Analysis (IPA) software (QIAGEN, Redwood City, Version 134816949) to generate biological insights from differential gene expression profiles in the thalamus. IPA’s Core Analysis module and its associated functionality were specifically used to identify Canonical pathways, Disease and Functions, and Regulator Effects to identify biological processes impacted by differently expressed genes (DEGs) in the thalamus as a result of cholestatic liver injury and after anti-TNF treatment.

## Quantitative RT-PCR

Quantitative Real-Time Reverse Transcription PCR (qRT-PCR) was used to validate the gene expression changes identified in our RNA-seq analyses. All PCR reactions were performed using PowerUp SYBR Green Master Mix on a QuantStudio 3 Real-Time PCR System (Thermo Fisher Scientific).

## Anti-TNF treatment

To delineate a potential role of TNF in cholestatic liver injury‑associated changes in the thalamic transcriptomic molecular signature, mice underwent BDL and then received intraperitoneal injections of either phosphate‑buffered saline (PBS) or anti‑TNF antibodies (300 µg) every other day, starting two days after surgery [23, 34]. Sham-operated controls received PBS alone on the same schedule (days 2, 4, 6, and 8). Brain and blood samples were collected on day 10 post‑surgery for subsequent PCR, RNA‑seq, and blood‑chemistry analyses.

### Statistical analysis

Data are presented as mean ± standard error of the mean (SEM). Statistical analyses were performed using GraphPad Prism software v10.4.1 (GraphPad Software Inc., San Diego, CA, USA). Normality was evaluated using the Kolmogorov–Smirnov algorithm in GraphPad Prism. If the data satisfied the normality assumption, we applied parametric tests (unpaired t-test or one-way ANOVA). When normality was not met, we used a non‑parametric Mann‑Whitney test. Statistical significance was defined as p *≤* 0.05.

## Results

### Bile duct ligation model of cholestasis

Successful induction of cholestatic liver injury was confirmed by identification of significant elevations in serum alanine aminotransferase (ALT), alkaline phosphatase (ALP), aspartate aminotransferase (AST), and total bilirubin (TBil) levels in BDL vs. sham control mice [22–25] Suppl. Figure 1.

### Cholestatic liver injury is associated with reduced thalamic volume

**Fig. 1:**
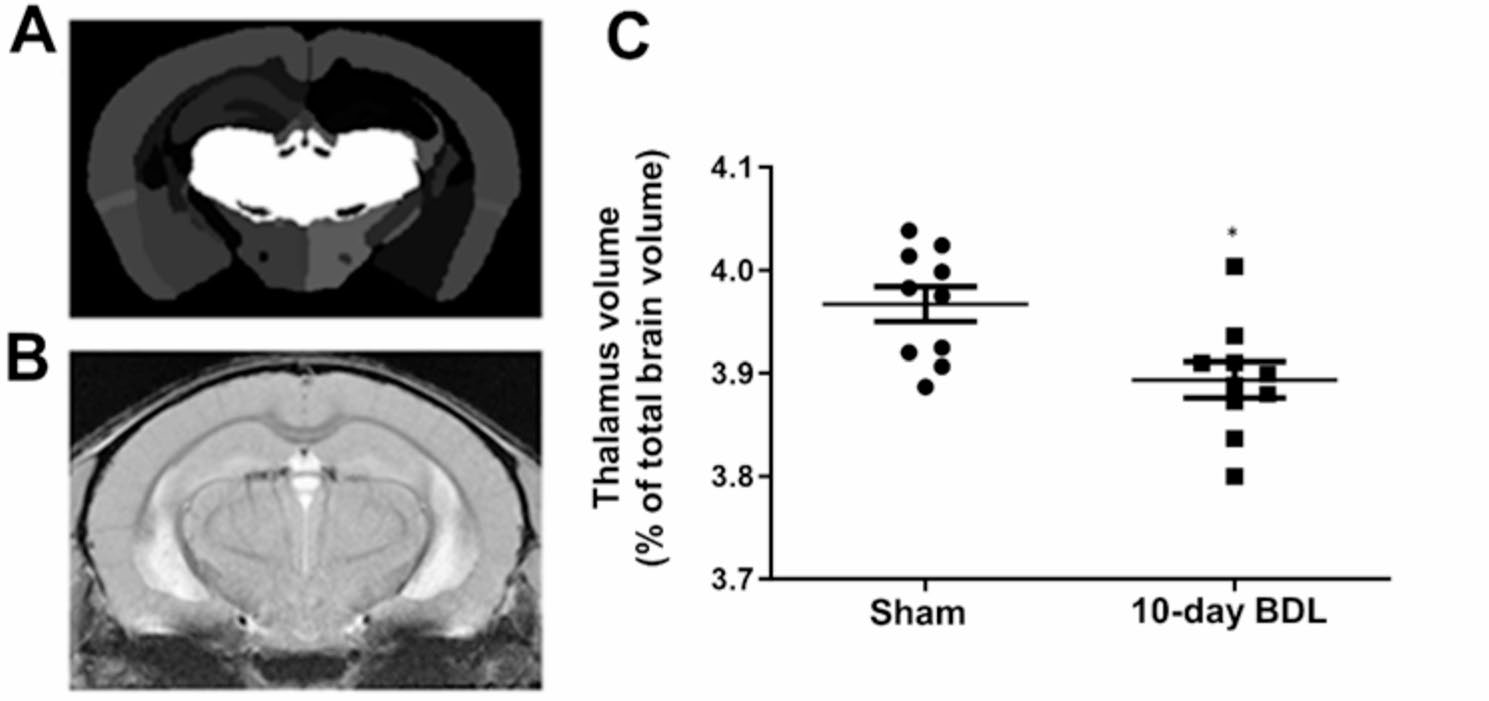
Magnetic resonance imaging (MRI) shows a significant reduction in thalamic volume in BDL vs. sham control animals. **A** Region of interest image generated after image co-registration to segmented mouse atlas. **B** FLASH image used as the anatomical reference image for co-registration (TE = 6.5ms, TR=1500ms, a = 60°, resolution = 0.375 × 0.375 × 0.25 mm³). **C** Thalamic volume as % of total brain volume in day 10 sham vs. BDL mice. *p = 0.0074, *n* = 10 mice per group

To investigate the impact of cholestatic liver disease on thalamic volume, bile duct-ligated and sham control mice were subjected to high-resolution in vivo MRI imaging 10 days post-surgery. Quantitative volumetric analysis revealed a significant reduction in thalamic volume, normalized to total brain volume, in BDL mice compared to sham controls (Fig. 1).

### Cholestatic liver injury induces molecular changes in the brain characterized by disrupted neural signaling and suppressed cellular proliferation and myelination pathways

**Fig. 2:**
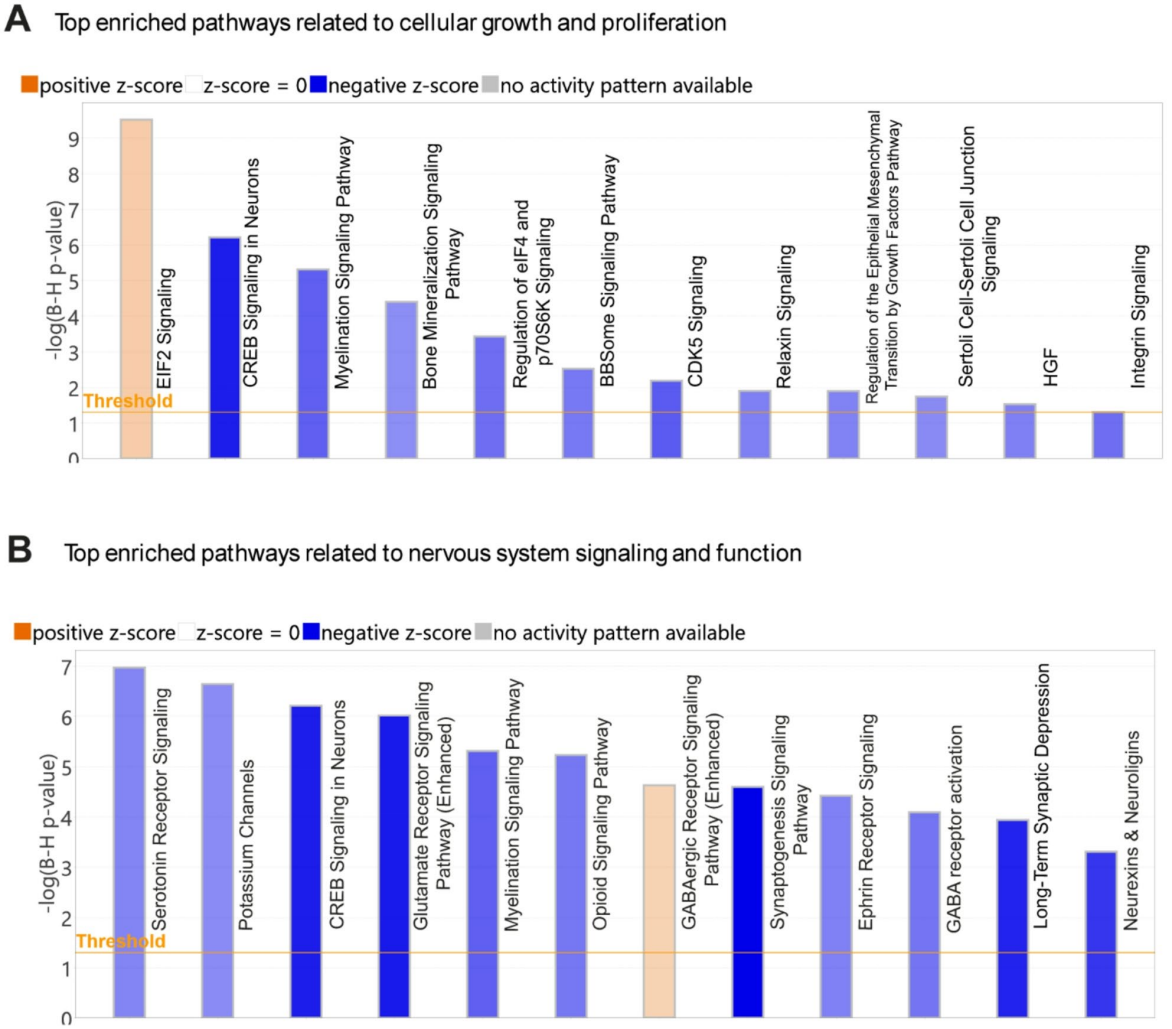
Top dysregulated pathways identified through a targeted enrichment analysis focused on brain‑specific signaling and cellular growth-proliferation in thalamic tissue of BDL vs. sham mice. Ingenuity Pathway Analysis (IPA) canonical pathways chart showing the top 12 significantly enriched biological pathways from gene expression data in the thalamus of BDL vs. sham mice that passed an analysis cutoff of adjusted p-value *≤* 0.05 and absolute activation z score of *≥* 2. The vertical bars represent different canonical pathways. The length of each bar correlates to the significance of enrichment expressed as -log (B-H p-value). The horizontal orange line represents the threshold cutoff for FDR-adjusted p-values of ≤ 0.05. Orange and blue shaded bars represent predicted pathway activation and inhibition, respectively. **A** IPA pathways chart displaying the top 12 enriched pathways related to cellular growth and proliferation. **B** IPA pathways chart displaying the top 12 enriched pathways related to nervous system signaling and function. A total of nine thalamic samples per treatment group were sequenced and subsequently combined for bulk RNA-seq analysis

BDL induced significant alterations in the thalamic tissue gene expression profile compared to sham controls, as visualized by a volcano plot (Suppl. Figure 2). All gene transcripts meeting predefined differential expression thresholds (FDR < 0.05, absolute fold change ≥ 1.2, and maximum group mean ≥ 1) are detailed in Suppl. File 1. To elucidate the potential biological significance of thalamic transcriptional changes, tissue DEG sets were analyzed using IPA Core Analysis which utilizes processed RNA-seq data and provides insights into pathways, regulators, and disease or function associations that can be linked to the DEG alterations. Key pathway analysis findings are summarized below and indicate BDL-associated changes in thalamic gene expression signatures linked to altered myelination, cellular growth, neural proliferation, and neurotransmission:

(i) Impaired myelination pathways: Canonical Pathway analysis, using IPA mapped thalamic DEGs onto established canonical pathways, predicted a BDL-associated inhibition of the *Myelination Signaling Pathway*, consistent with inhibition of myelination processes (Fig. 2A, B). A potential deficit in myelination in BDL vs. sham control mice was further supported by a significant reduction in thalamic PLP1 (Proteolipid Protein 1) mRNA levels which encodes for a protein essential for myelin integrity and function (Suppl. Figure 3).

(ii) Inhibition of cellular growth and proliferation pathways: RNA‑seq profiling and IPA analysis of the thalamus revealed tissue level gene expression signatures consistent with suppressed cellular growth and reduced neurogenesis pathways in BDL vs. sham mice. A targeted Canonical Pathways enrichment analysis specifically focused on pathways governing Cell Cycle Regulation, Cellular Growth, Proliferation and Development, and Growth Factor Signaling, identified significant alterations in several key pathways in the thalamus of BDL mice compared with sham controls that are essential for normal thalamic neuronal growth, differentiation, survival, and plasticity, including predicted inhibition of *CDK5 Signaling*, *CREB Signaling in Neurons*, *BBSome Signaling Pathway*, and *Regulation of eIF4 and p70S6K Signaling*, and predicted activation of EiF2 signalling (Fig. 2A**)**. File 2 in Supplementary Material provides a complete list of significantly impacted pathways (FDR ≤ 0.05).

Additionally, the Diseases and BioFunctions analysis module of IPA, which maps gene‑expression changes to predicted biological outcomes, revealed significant enrichment with an overall predicted inhibition of biofunctions linked to proliferation of neural cells, development of neural cells, and growth of neurites in the thalamus of BDL vs. sham control mice. Table 1 lists the highest‑ranking disease or function annotations together with their predicted activation states. A complete list of all enriched terms under the “Nervous System Development and Function” and “Cellular Growth and Proliferation” categories meeting predefined criteria (FDR‑adjusted *p* < 0.05 and absolute predicted activation Z‑score ≥ 2), along with molecules associated with each term, is provided in Supplemental File 3.

**Table 1:** Top enriched diseases and biofunctions identified by IPA Analysis in the thalamus of BDL vs. Sham mice. Table 1 lists the top identified diseases or functions under the “Nervous System Development and Function” and “Cellular Growth and Proliferation” categories that passed an enrichment cutoff of an FDR-adjusted p-value *≤* 0.05 and an absolute predicted activation Z-score ≥ 2. The last column in the table indicates the number of differentially expressed genes from our dataset that overlap with the network associated with each disease or function annotation. Nine thalamic samples per treatment group were sequenced with bulk RNA-seq analysis

Diseases or functions annotation	B-H *p*-value	Predicted activation state	Activation z-score	# Molecules
Development of neural cells	2.81E-37	Decreased	-3.374	343
Development of central nervous system	3.06E-23	Decreased	-3.162	233
Morphogenesis of neurons	5.68E-25	Decreased	-3.12	251
Development of neurons	1.49E-33	Decreased	-3.109	323
Neuritogenesis	2.47E-24	Decreased	-3.034	247
Proliferation of neuronal cells	5.12E-20	Decreased	-2.667	176
Neurotransmission	8.64E-17	Decreased	-2.141	143
Growth of neurites	4.68E-15	Decreased	-3.013	142
Proliferation of neural cells	2.21E-23	Decreased	-3.415	227

**Fig. 3:**
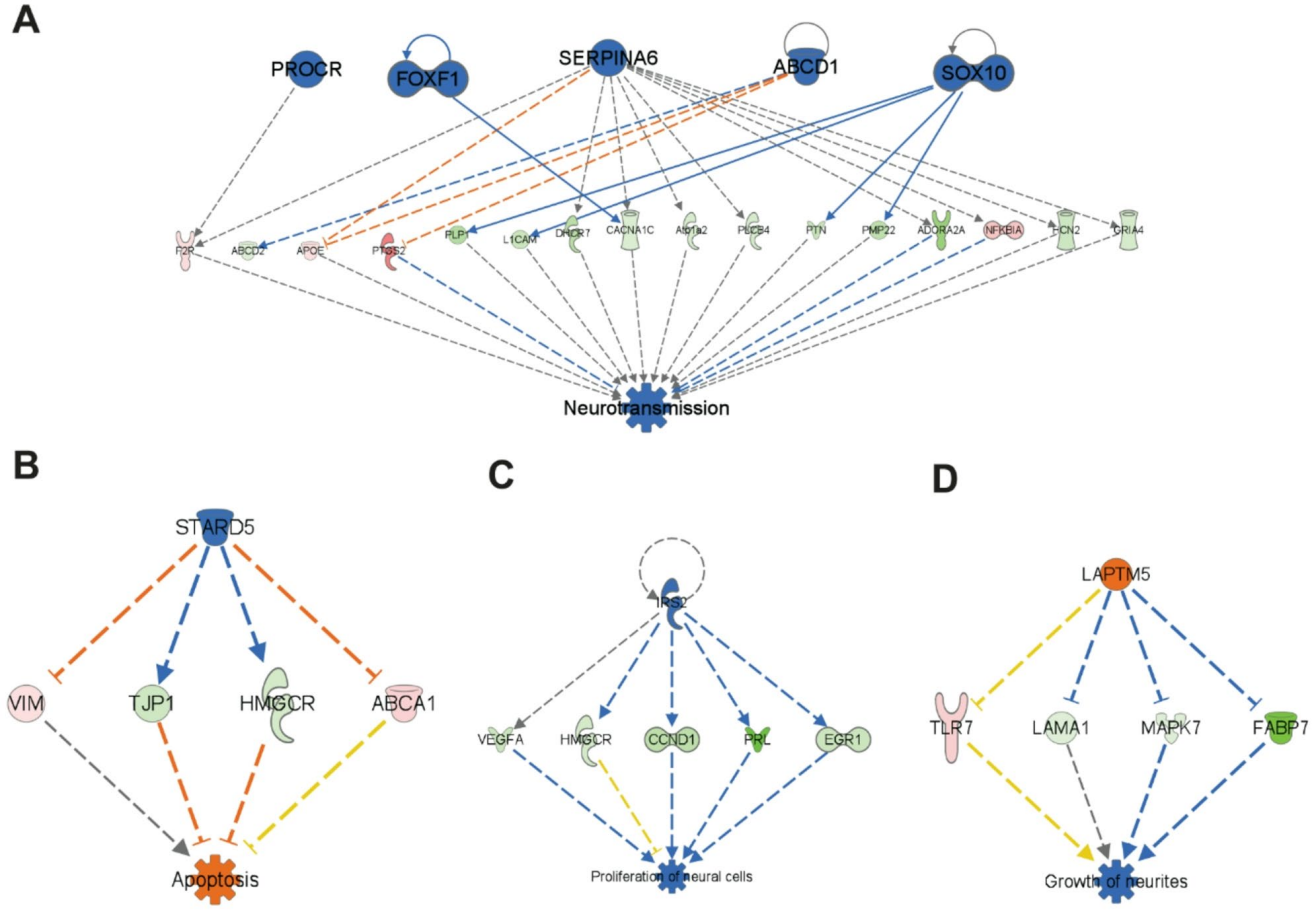
Selected networks generated from IPA regulator effects analysis for thalamic DEGs of BDL vs. sham mice. Regulator effects analysis using IPA was performed on thalamic transcriptome datasets comparing BDL vs. sham mice (**A**, **B**, **C**, and **D**). Upstream regulators are shown in the top tier and are predicted to be either activated (orange color) or inhibited (blue color) in the thalamus, based on an absolute z-score threshold of 2.0 and a p-value cutoff of 0.05. The target molecules (DEGs) that connect upstream regulators to downstream biological functions are displayed in the middle tier. These genes are color-coded as upregulated (red) or downregulated (green) and shaded to reflect varying expression levels. In the bottom tier, downstream diseases and functions that are predicted to be impacted by this directional network are shown. Significance is defined by the same z-score and p-value thresholds (as above). Predicted activation is indicated in orange, while inhibition is shown in blue. Solid lines indicate direct relationships, and dashed lines indicate indirect relationships. Genes in the figure are represented by standard gene short form symbols - the corresponding full names of these genes can be found in Suppl. File 5. Genes represented are indicated by standard gene symbols. The corresponding official full gene names are shown in Suppl. File 4. A total of nine thalamic samples per treatment group were sequenced and subsequently combined for bulk RNA-seq analysis.

Consistent with pathway-level thalamic gene expression dysregulation identified using IPA, direct measurement of classical cell proliferation gene expression markers using qRT-PCR showed significantly decreased thalamic mRNA expression levels of the cell proliferation marker Ki67 and increased mRNA expression of the cell-cycle inhibitor Cdkn1a (p21) in BDL mice compared to controls (Suppl. Figure 3).

(iii) Altered neurotransmission: Thalamic neurotransmitter networks critically regulate behavior, including motivation-driven behavior closely linked to the development of central fatigue [11, 35]. IPA analysis of RNA‑seq data indicated significant dysregulation within key molecular pathways governing thalamic neurotransmission and neural function. Specifically, targeted enrichment analysis of brain-specific signaling pathways revealed significant predicted inhibition in several key pathways essential for normal thalamic synaptic transmission and network excitability in BDL mice compared to sham control mice, including the *Serotonin Receptor Signaling Pathway*, *Potassium Channels*, *Neurexins and Neuroligins*, and the *Glutamate Receptor Signaling Pathway*. In contrast, the inhibitory *GABAergic Receptor Signaling Pathway* was predicted to be activated (Fig. 2B). These pathway-level gene expression profile alterations provide a molecular framework that could lead to disrupted thalamic neural processing in the context of cholestatic liver disease.
(iv) Regulator Effects Analysis-generated causal hypotheses connecting gene expression changes in the thalamus of BDL mice to reduced neural cell proliferation and suppressed neurotransmission: Regulator Effects Analysis was performed on RNA-seq data using IPA to connect and merge upstream regulators with disease and function results, using thalamic DEGs in our dataset as intermediary molecules. This approach generates causal hypotheses and produces directional molecular networks that predict the activation or inhibition of downstream biological functions or diseases based on our input data. This analysis identified several molecular networks whose directional activities may contribute to a reduction in thalamus size and altered neurotransmission in BDL mice. These changes include biological networks predicted to inhibit neural cell proliferation and growth of neurites, to enhance apoptosis, and to suppress neurotransmission **(**Fig. 3 and Suppl. File 3).

### Systemic anti‑TNF treatment attenuates dysregulated BDL-associated changes in thalamic molecular pathways and networks related to neuronal communication, cell proliferation, and cellular growth pathways

To define a potential role of systemic TNF signaling in driving BDL‑associated transcriptomic dysregulation in the thalamus, BDL mice received intraperitoneal injections of either PBS or anti‑TNF neutralizing antibodies every other day, starting two days post-surgery. Sham-operated controls received PBS on the same schedule (days 2, 4, 6, and 8). Importantly, similar to our previously published findings, anti-TNF treatment did not alter the severity of BDL-associated liver injury [23] (Suppl. Figure 1).

**Fig. 4:**
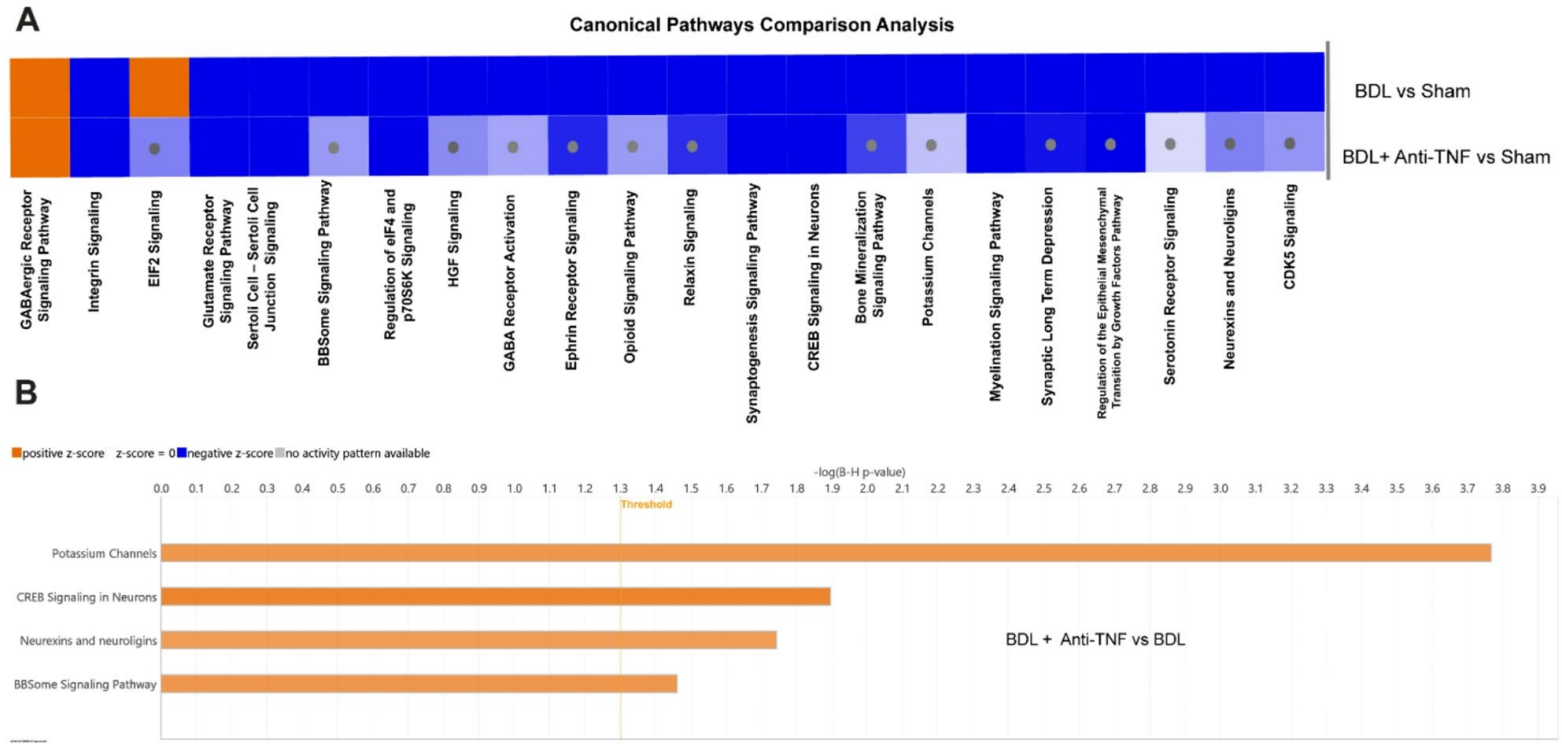
Anti-TNF treatment attenuates key pathway dysregulation in the thalamus of BDL mice. The comparison analysis function in IPA allows visual comparison between various comparison analysis sets side by side. **A** The canonical pathways heatmaps generated by the comparison analysis tool and visualized with Z scores for thalamic pathway activity for BDL vs. sham and BDL + TNF vs. sham mice indicate that anti-TNF prevents the impact of BDL on key thalamic gene expression signatures linked to altered proliferation and neurotransmission pathways in BDL mice. Shades of orange and blue in the heat map indicate predicted pathway activation and inhibition, respectively (Gray dots indicate that the activity Z score did not pass the significance cutoff level of 2 for pathways shown in the BDL+ anti-TNF vs. sham comparison row, but were significant in the BDL vs. sham comparisons shown in the row above). **B** The IPA pathway chart displays enriched pathways associated with cellular growth, proliferation, and nervous system signaling and function in the thalamus of the BDL group that are significantly activated (Z score) by anti-TNF treatment (orange colour) compared to activation changes of these pathways in BDL mice without anti-TNF treatment. All listed pathways passed the predefined analysis threshold of FDR ≤ 0.05 and an absolute activation z-score of ≥ 2. The horizontal bars represent different canonical pathways, and the length of each bar correlates to the significance of enrichment expressed as -log (B-H p-value). Orange and blue bars represent predicted pathway activation and inhibition, respectively. The horizontal vertical orange line represents the threshold cutoff for p-values of ≤ 0.05. Nine thalamic samples for the BDL and sham groups underwent bulk RNA-seq analysis. Four thalamic samples were used for the BDL + anti-TNFα group RNA-seq analysis

The IPA Canonical Pathways tool was used to determine the impact of systemic TNF neutralization on thalamic DEG expression signatures for biological pathways that we had shown were significantly dysregulated in the thalamus of BDL vs. sham control mice. Pathway enrichment analysis filtered to specifically include thalamic pathways linked to brain signaling, cellular growth, and proliferation (significance cutoff of FDR ≤ 0.05 and absolute activation z-score of ≥ 2) is shown in Fig. 4. A comprehensive list of all significantly enriched pathways meeting these criteria is provided in Suppl. File. 2. Anti-TNF treatment exerted a directionally consistent effect on critical thalamic pathways in BDL mice with gene expression patterns indicative of anti-TNF treatment-induced activation of neurotransmission, homeostasis, cellular survival, and growth.

A comparison analysis gene expression heatmap of the Canonical Pathway analysis presented in Fig. 4A indicates that many significant BDL-related inhibitory effects on key thalamic functional pathways are prevented by anti-TNF treatment (indicated by gray dots overlayed on corresponding pathways in BDL+anti-TNF vs. sham row in the heat map), resulting in a net predicted increase in activity of these pathways compared to the transcriptome profile of BDL mice that did not receive anti-TNF (Fig. 4A, B). Key anti-TNF ‘rescued’ dysregulated thalamic pathways include *CREB Signaling in Neurons*, *Neurexins and Neuroligins*, *Potassium Channels*, and the *BBSome Signaling Pathway* (Fig. 4B).

The Diseases and BioFunctions Analysis tool was used to explore the impact of anti-TNF treatment on molecular networks associated with ‘Nervous System Development and Function’ and ‘Cellular Growth and Proliferation’ categories. Anti-TNF treatment impacted expression of key molecules involved in neural signaling, cell proliferation, neuronal survival, and synaptic plasticity in the thalamus of BDL mice. Collectively, the directionality and pattern of changes in disease- and function- related networks impacted by anti-TNF treatment suggest a systemic TNF-mediated thalamic functional and growth pathway inhibition in BDL mice. Table 2 shows the top thalamic *Diseases and Biofunctions* modulated by anti-TNF treatment in BDL mice. A complete list of all enriched diseases and functions under the “Nervous System Development and Function” and “Cellular Growth and Proliferation” categories passing our enrichment cutoff (FDR-adjusted p-value < 0.05 and absolute predicted activation Z-score ≥ 2), along with a detailed list of all associated molecules annotated with each disease and function, is provided in Suppl. File. 3. Consistent with a beneficial effect of anti-TNF treatment on neural proliferation molecular pathways in the thalamus of BDL mice, qRT-PCR analysis revealed an upregulation of mRNA expression for the cellular proliferation marker Ki‑67 in the thalamus of BDL mice with TNF neutralization (Suppl. Figure 4).

**Table 2:** Top Ingenuity Pathway Analysis Diseases and Biofunctions in the BDL + anti-TNF vs. BDL thalamus. The table lists the top identified diseases or functions under the “Nervous System Development and Function” and “Cellular Growth and Proliferation” categories impacted by anti-TNF treatment in BDL mice. The last column in the table indicates the number of differentially expressed genes from our dataset overlapping with the identified network of each disease or function annotation. Nine thalamic samples for the BDL and sham groups underwent bulk RNA-seq analysis. Four thalamic samples were used for the BDL + anti-TNFα group RNA-seq analysis

Diseases or functions annotation	B-H *p*-value	Predicted activation state	Activation z-score	# Molecules
Development of neurons	5.46E-10	Increased	2.581	43
Neuritogenesis	1.03E-09	Increased	2.541	37
Excitatory postsynaptic potential	4.78E-09	Increased	2.433	15
Neurotransmission	3.90E-15	Increased	2.179	34
Synaptic transmission	7.02E-12	Increased	2.129	27
Neuroprotection	2.74E-05	Increased	2.046	8
Formation of dendritic spines	5.14E-04	Increased	2.646	9
Branching of neurites	5.34E-04	Increased	2.587	16
Proliferation of neural cells	2.57E-06	Increased	2.153	29
Branching of neurons	2.20E-04	Increased	2.774	17

**Fig. 5:**
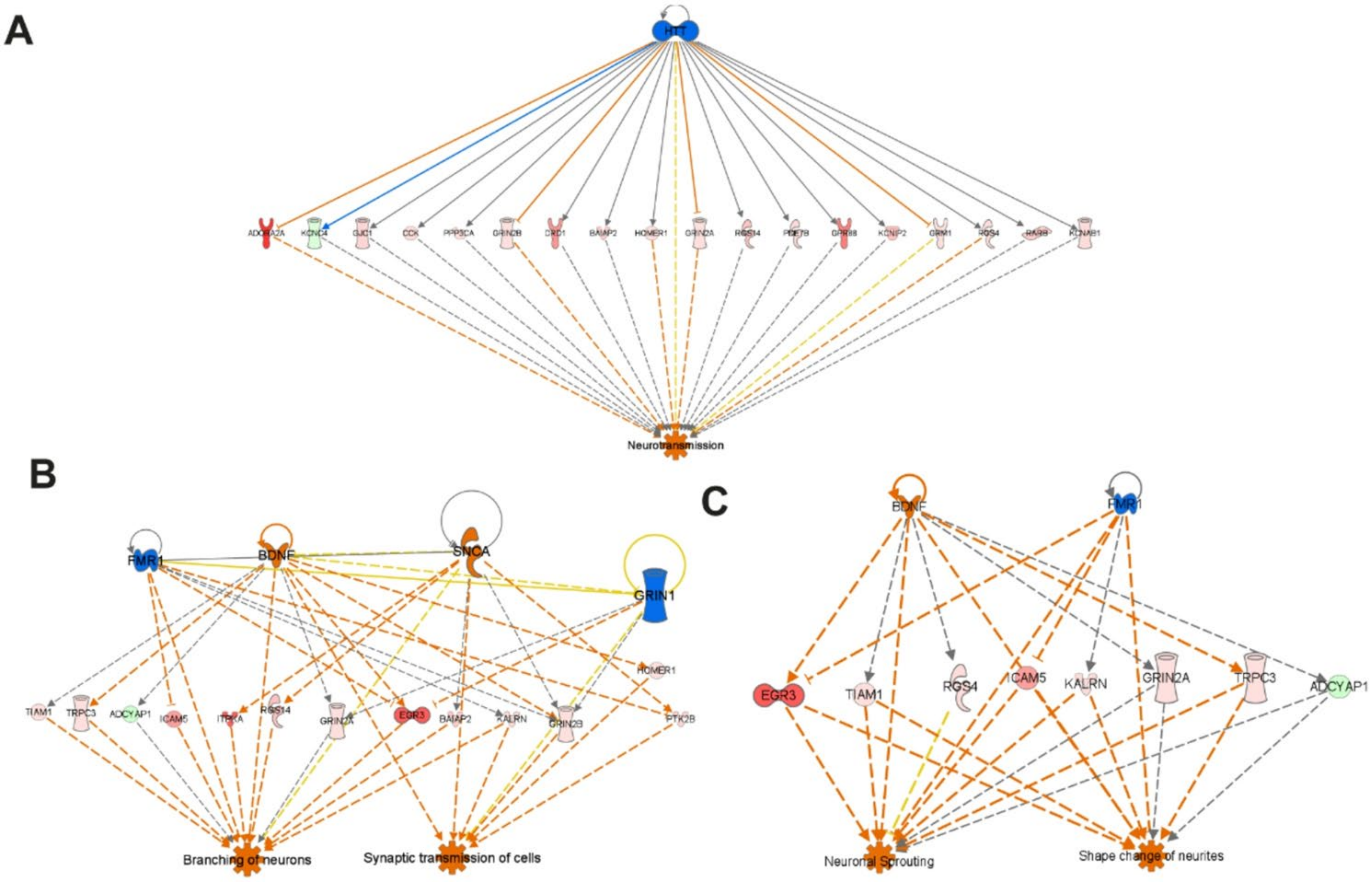
Selected regulator effects analysis networks for the DEGs in the thalamus of BDL+ anti-TNF vs. BDL mice. Selected Regulator Effects networks illustrating the impact of anti‑TNF treatment on thalamic gene expression signatures and associated biological processes, functions, or diseases in BDL mice (**A**,** B**,** C**). The top tier lists upstream regulators predicted to be activated (orange) or inhibited (blue) based on an absolute z-score of ≥ 2.0 and a p-value of ≤ 0.05. The middle tier displays treatment-responsive target genes (i.e., those altered by anti-TNF treatment in BDL mice vs. BDL alone) that bridge these regulators to downstream functions; upregulated genes are shown in shades of red, and downregulated genes are in shades of green. The bottom tier shows diseases and biological functions predicted to be affected by anti-TNF treatment in BDL mice; orange indicates predicted activation, and blue indicates predicted inhibition. Solid lines depict direct relationships, whereas dashed lines denote indirect relationships. Genes in the figure are represented by standard Gene symbols, and the corresponding full names of these genes can be found in Suppl. File 5. Genes are represented by standard gene symbols and corresponding official full gene names are in Suppl. File 4. Nine thalamic samples for the BDL and sham groups underwent bulk RNA-seq analysis. Four thalamic samples were used for the BDL + anti-TNFα group RNA-seq analysis

IPA’s Regulator Effects Analysis was employed to investigate how anti-TNF treatment could potentially modulate thalamic neurobiological and cellular processes in BDL mice. This hypothesis-generating approach leverages anti-TNF treatment-altered genes in the BDL thalamus as molecular intermediaries to generate predictions of potential effects these changes would be expected to have on diseases and functions, capitalizing on the large, pre-constructed, evidence-based networks contained within the IPA knowledge base. Using this tool, anti-TNF treatment in BDL mice was predicted to enhance processes that regulate neurotransmission and synaptic transmission, as well as enhance branching of neurons, neuronal sprouting, and shape changes in neurites (Fig. 5 and Suppl. File 4).

**Fig. 6:**
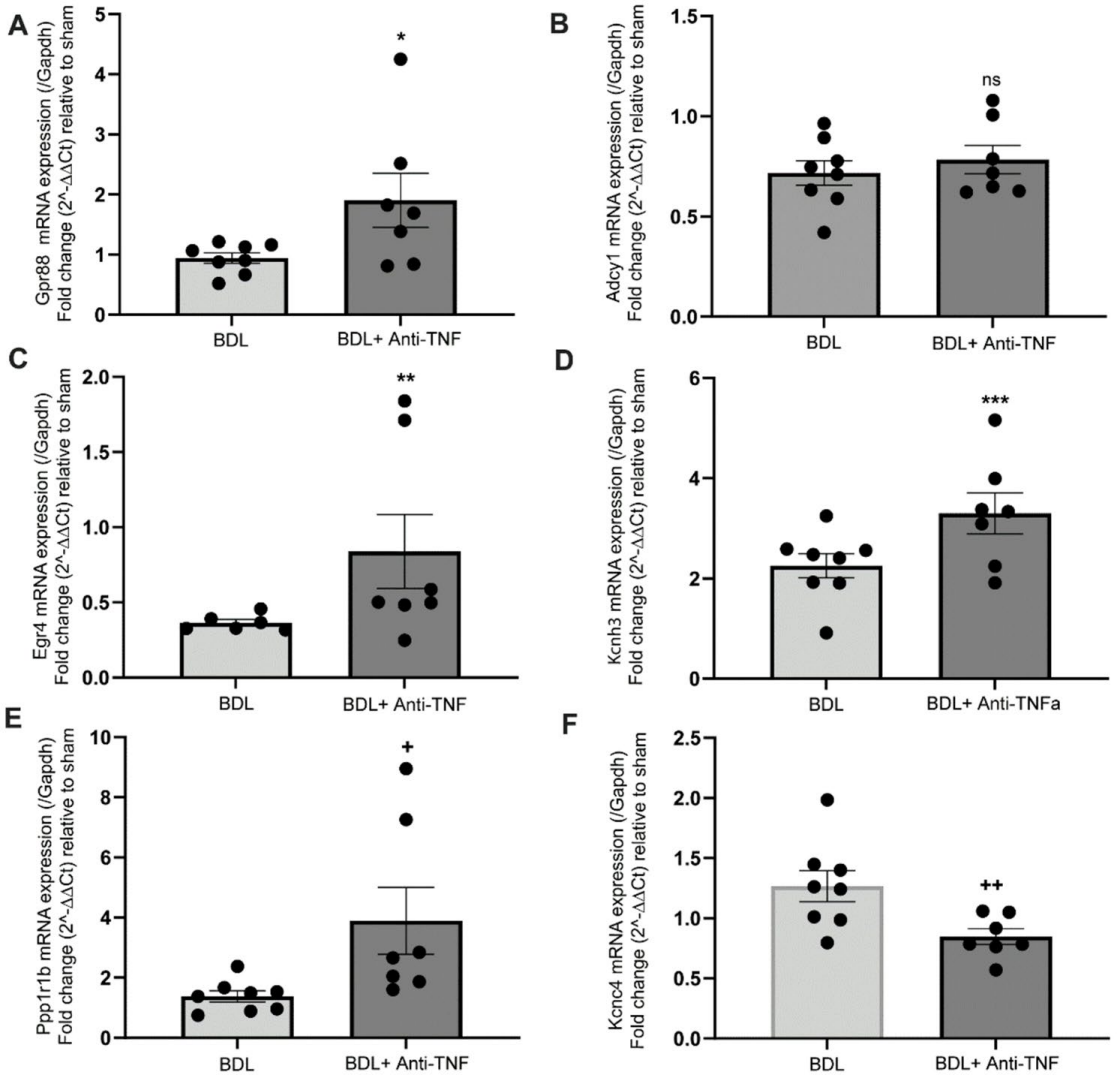
Quantitative RT-PCR confirmation of anti-TNF-mediated changes in expression levels for key BDL-dysregulated genes determined using RNA-seq. Panels **A**-**F** show qRT-PCR mRNA expression results for selected genes comparing expression levels in BDL+ anti-TNF vs BDL without anti-TNF (normalized to expression levels in sham thalamus). N = 7 and 8 mice per group. Symbols *, **, ***, +, ++ represent P-values of 0.0434, 0.035, 0.041, 0.0336, and 0.0154 respectively. For ADCy1, the result was not significant, with a p-value of 0.4833. Genes are represented by standard gene symbols and corresponding official full names are in Suppl. File 5

In contrast to potential beneficial effects of blocking systemic TNF signaling on BDL-associated changes in thalamic regulatory pathways, as outlined above, anti-TNF treatment did not prevent dysregulation of thalamic myelination pathways associated with BDL as assessed using RNA-seq and IPA analysis (or qRT-PCR for PLP1 mRNA expression; Suppl. Figure 4). RNA-Seq results were further validated using qRT-PCR to confirm both the direction and magnitude of gene expression changes observed in the RNA-Seq data for a selection of genes associated with key dysregulated thalamic pathways in BDL mice, as well as those restored by anti-TNF treatment (Fig. 6).

## Discussion

In PBC, extrahepatic symptoms including cognitive impairment, altered mood, and fatigue, often overshadow biochemical markers used for monitoring disease progression and treatment response, posing a complex clinical challenge and directly impacting patient quality of life [36–38]. The complex, poorly understood nature of symptom development in PBC underscores the urgent, unmet medical need for targeted treatments that address symptoms, and highlights the common disconnect between liver disease-directed therapies and improvements in patient quality of life.

Resting state functional and structural MRI can be used to delineate the impact of neurological and systemic inflammatory disease upon specific brain regions, and on neural communication within and between brain regions that form networks to regulate normal behavior [39]. Using MRI-based approaches in patients, it has become clear that the thalamus plays a critical role as a neural integration hub regulating communication between higher cortical brain centers and subcortical structures within the limbic system (the ‘emotional brain’) and striatum (including the basal ganglia) [40]; brain regions that critically regulate complex behaviors such as motivation, reward, alertness, arousal, and attention that are adversely impacted in the context of central fatigue [11, 41]. Importantly, structural and functional alterations in the thalamus are associated with disease-associated symptoms, including fatigue, in many chronic illnesses, including multiple sclerosis [12], neuromyelitis optica spectrum disorder (NMOSD) [42], concussion [43], long-Covid [13, 15], and stroke [44]. Therefore, it is plausible that changes in thalamic structure and/or function could play an important role in the development of these symptoms in PBC patients. Indeed, in previous work by us and others PBC patients showed robust changes in the thalamus [1, 16, 18] linked to symptom severity [1, 18]. However, the etiological basis for these changes remains unknown, but is of significant importance if we hope to develop specific, targeted approaches that effectively treat adverse symptoms in these patients.

To mechanistically address this knowledge gap we used a well-characterized mouse model of cholestatic liver disease that we have previously shown reproducibly generates changes in brain neurotransmission linked to reduced motivation and social interaction behaviors that mimic many adverse behaviors commonly reported in PBC patients [22–25, 33]. Using structural MRI, we now report that BDL mice exhibit a reduction in thalamic volume, similar to that observed in PBC patients [18]. Importantly, thalamic volume reductions of similar magnitude have consistently been documented in many neurological disorders and were linked to altered behaviors, including fatigue and impaired cognition [12, 15, 42, 43]. However, mechanisms leading to reduced thalamic volume remain unclear. Normal thalamic volume is determined mainly by neuronal and glial cell populations, and myelin content. A number of genetic alterations are associated with reduced thalamic volume, and in turn to a diverse array or psychiatric and neurological disorders [45]. Moreover, neuroinflammation can lead to damage of neurons and their structural components within the CNS, including axons, synapses and dendritic projections, and inhibit myelination processes [46]. Our RNA-seq analyses identify significant disruption of the thalamic gene signature in cholestatic mice that involves multiple physiological pathways known to critically regulate neuronal survival (e.g., CREB signaling in neurons, CDK5 signaling, EiF2 signaling) [47], synapse formation and function (Neurexins and neuroglins) [48], synaptogenesis [49], and myelination [50]. Furthermore, regulator effects analysis of BDL-associated DEGs predicted suppression of proliferation of neuronal cells and enhanced apoptosis in the thalamus. These findings in our BDL model parallel MRI findings reported in PBC patients of a decreased thalamic apparent diffusion coefficient indicating reduced neuronal density and myelination [16], suggesting similar molecular processes may be driving thalamic volume reductions in both PBC and our animal model.

Spontaneous neural activity in specific brain regions is commonly inferred from resting state functional MRI (rsfMRI) studies by measuring amplitude of low frequency fluctuations (ALFF) [51]. We have previously identified reduced ALFF in the thalamus of PBC patients [18], suggesting intrinsic thalamic neural dysfunction in PBC, likely reflecting changes in neurotransmission. Neurotransmitter microcircuitry within the thalamus is characterized predominantly by excitatory (i.e., glutaminergic) neurons, and a smaller population of inhibitory (i.e., GABAergic) interneurons, whose function can be further regulated by a number of neuromodulatory processes mediated by metabotropic glutamate receptors, dopamine, serotonin, endogenous opioids, and other neuropeptides [52]. In our current study, we identified thalamic DEG changes in cholestatic mice involving many nervous systems signaling and function pathways known to critically regulate neurotransmission, including significant inhibition of pathways regulating serotonergic receptor signaling, potassium channels, glutamate receptor signaling, opioid signaling, ephrin receptor signaling, and GABAergic receptor signaling. These changes in key pathways regulating neurotransmission would be expected to significantly impact thalamic neural networks regulating behavior, similar to observations in multiple sclerosis patients [53].

Alterations in ALFF measurements are often analyzed in rsfMRI studies in conjunction with measures of neural functional connectivity to understand how findings in a given brain region impact neural communication with other brain regions and neural networks to ultimately change behavior (termed resting state functional connectivity [rsFC]) [54]. For the thalamus, extensive reciprocal neural connections with the cortex, amygdala and striatum maintain normal behavior, including the regulation of motivational and reward responses [55, 56]. In previous work using rsfMRI we identified significant changes in functional connectivity between the thalamus and other brain regions in PBC patients. Specifically, PBC patients showed increased rsFC between the thalamus and the putamen (part of the basal ganglia), hippocampus, amygdala and the motor and sensory cortex regions [1], key behavior-regulating brain regions.

A key issue that remains is, how does immune-mediated liver injury cause changes in thalamic structure and function in PBC patients to alter behavior? Systemic inflammation is relayed to the CNS via a number of pathways and leads to activation of microglia, neuroinflammatory responses, and altered behavior, including fatigue [20, 21, 57]. This process can be readily demonstrated in healthy volunteers treated with endotoxin to activate systemic immunity [58], and in animal models [57]. Moreover, low grade systemic inflammation in healthy volunteers is associated with decreased thalamic volume [59]. In our animal model we have previously documented a key role of circulating TNF in signaling the brain and activate microglia, leading to altered neurotransmission and reduced motivational behaviour and social withdrawal [23, 24]. Therefore, in our current study we inhibited systemic TNF signaling in BDL mice to determine the impact on thalamic gene expression signatures and pathways that were dysregulated in cholestatic mice. Indeed, we found that inhibition of TNF signaling in BDL mice mitigated or attenuated BDL-associated changes in many of these pathways. Specifically, BDL-related inhibition of thalamic *CDK5 signaling*, *neurexins and neuroligins*, *potassium channels*, and *BBsome signaling* pathways were all significantly attenuated. Thalamic regulator effects analysis further supported a beneficial impact of TNF inhibition in BDL mice on thalamic neuronal processes and function, including predicted activation of neurotransmission and synaptic transmission as well as branching of neurons, neuronal sprouting, and shape change of neurites.

In this study, blocking the effects of systemic TNF using a specific neutralizing antibody did not alter BDL-associated liver inflammation. This finding is consistent with our previously published findings in this model [23, 24]. Although some previous studies in BDL mice using a gene knockout (KO) approach have suggested that the absence of TNF protects against liver injury [60], other studies using the same approach found that TNF deficiency did not alter liver injury or inflammation [61]. The lack of an impact of systemic TNF neutralization on markers of BDL-associated liver injury in all our studies to date are consistent and suggests that systemic TNF signals the brain to alter thalamic gene expression signatures independent of an effect of liver disease severity.

While transcriptomic and anatomical findings suggest impaired cellular and neurotransmission pathways in our BDL model, in a brain area that has been linked closely in humans to the regulation of numerous behaviors, direct causation cannot be inferred from our current data. Moreover, in our current work functional assessments of fatigue were not performed. We acknowledge the limitations of this work, particularly the inability to directly link thalamic changes observed in our animal model to altered behaviors such as fatigue. This study was designed to examine structural and functional alterations in the thalamus in the context of cholestatic liver injury, with comparison to changes reported in patients with PBC, rather than to establish a direct causal link to symptoms including fatigue. Future work using complementary behavioral approaches will be needed to confirm the mechanistic role of these pathways in inducing a fatigue-like behavioural phenotype and to define the impact of TNF on fatigue-related neural and behavioral processes.

## Supplementary Information

Below is the link to the electronic supplementary material.

Supplementary Material 1

Supplementary Material 2

Supplementary Material 3

Supplementary Material 4

Supplementary Material 5

Supplementary Material 6

## Data Availability

Data supporting the results reported in this manuscript are available upon request.

## Abbreviations

ALFF: Amplitude of low–frequency fluctuations
ALP: Alkaline phosphatase
ALT: Alanine aminotransferase
AST: Aspartate aminotransferase
CNS: Central nervous system
DEGs: Differentially expressed genes
EIC: Experimental imaging centre
FDR: False discovery rate
FLASH MRI: Fast low–angle–shot magnetic resonance imaging
IPA: Ingenuity Pathway Analysis
MRI: Magnetic resonance imaging
PBC: Primary biliary cholangitis
PBS: Phosphate–buffered saline
qRT: PCR–quantitative real–time reverse–transcription PCR
rsFC: Resting–state functional connectivity
rsfMRI: Resting–state functional MRI
TBil: Total bilirubin
TNF: Tumour necrosis factor
UDCA: Ursodeoxycholic acid

## References

[1] Mosher VAL, Swain MG, Pang JXQ, Kaplan GG, Sharkey KA, MacQueen GM, et al. Primary Biliary Cholangitis Alters Functional Connections of the Brain’s Deep Gray Matter. Clin Transl Gastroenterol. 2017;8(7):e107. 28749455 10.1038/ctg.2017.34 PMC5539342

[2] Jopson L, Jones DE. Fatigue in Primary Biliary Cirrhosis: Prevalence, Pathogenesis and Management. Dig Dis. 2015;33(Suppl 2):109–14. 26641884 10.1159/000440757

[3] Goldblatt J, Taylor PJ, Lipman T, Prince MI, Baragiotta A, Bassendine MF, et al. The true impact of fatigue in primary biliary cirrhosis: a population study. Gastroenterology. 2002;122(5):1235–41. 11984509 10.1053/gast.2002.32993

[4] Carbone M, Mells GF, Pells G, Dawwas MF, Newton JL, Heneghan MA, et al. Sex and age are determinants of the clinical phenotype of primary biliary cirrhosis and response to ursodeoxycholic acid. Gastroenterology. 2013;144(3):560–9. e7; quiz e13-4. 23246637 10.1053/j.gastro.2012.12.005

[5] Dyson JK, Wilkinson N, Jopson L, Mells G, Bathgate A, Heneghan MA, et al. The inter-relationship of symptom severity and quality of life in 2055 patients with primary biliary cholangitis. Aliment Pharmacol Ther. 2016;44(10):1039–50. 27640331 10.1111/apt.13794 PMC5082554

[6] Carbone M, Bufton S, Monaco A, Griffiths L, Jones DE, Neuberger JM. The effect of liver transplantation on fatigue in patients with primary biliary cirrhosis: a prospective study. J Hepatol. 2013;59(3):490–4. 23628322 10.1016/j.jhep.2013.04.017

[7] Zhang Y, Chen H-x, Shi Z-y, Du Q, Wang J-c, Wang X-f, et al. Brain structural and functional connectivity alterations are associated with fatigue in neuromyelitis optica spectrum disorder. BMC Neurol. 2022;22(1):235. 35761294 10.1186/s12883-022-02757-4 PMC9235096

[8] Hwang K, Bertolero MA, Liu WB, D’Esposito M. The Human Thalamus Is an Integrative Hub for Functional Brain Networks. J Neurosci. 2017;37(23):5594–607. 28450543 10.1523/JNEUROSCI.0067-17.2017 PMC5469300

[9] Zhang D, Snyder AZ, Shimony JS, Fox MD, Raichle ME. Noninvasive functional and structural connectivity mapping of the human thalamocortical system. Cereb Cortex. 2010;20(5):1187–94. 19729393 10.1093/cercor/bhp182 PMC2852505

[10] Shine JM. The thalamus integrates the macrosystems of the brain to facilitate complex, adaptive brain network dynamics. Prog Neurobiol. 2021;199:101951. 33189781 10.1016/j.pneurobio.2020.101951

[11] Shine JM, Lewis LD, Garrett DD, Hwang K. The impact of the human thalamus on brain-wide information processing. Nat Rev Neurosci. 2023;24(7):416–30. 37237103 10.1038/s41583-023-00701-0 PMC10970713

[12] Capone F, Collorone S, Cortese R, Di Lazzaro V, Moccia M. Fatigue in multiple sclerosis: The role of thalamus. Mult Scler. 2020;26(1):6–16. 31138052 10.1177/1352458519851247

[13] Leitner M, Opriessnig P, Ropele S, Schmidt R, Leal-Garcia M, Fellner M, et al. Changes in thalamic functional connectivity in post-Covid patients with and without fatigue. NeuroImage. 2024;301:120888. 39419425 10.1016/j.neuroimage.2024.120888

[14] Bungenberg J, Hohenfeld C, Costa AS, Heine J, Schwichtenberg K, Hartung T, et al. Characteristic functional connectome related to Post-COVID-19 syndrome. Sci Rep. 2024;14(1):4997. 38424415 10.1038/s41598-024-54554-3 PMC10904373

[15] Heine J, Schwichtenberg K, Hartung TJ, Rekers S, Chien C, Boesl F, et al. Structural brain changes in patients with post-COVID fatigue: a prospective observational study. EClinicalMedicine. 2023;58:101874. 36873426 10.1016/j.eclinm.2023.101874 PMC9969172

[16] Grover VP, Southern L, Dyson JK, Kim JU, Crossey MM, Wylezinska-Arridge M, et al. Early primary biliary cholangitis is characterised by brain abnormalities on cerebral magnetic resonance imaging. Aliment Pharmacol Ther. 2016;44(9):936–45. 27604637 10.1111/apt.13797 PMC5082539

[17] Kobal L, Surlan Popovic K, Avsenik J, Vipotnik Vesnaver T. ADC values as a biomarker of fetal brain maturation. Radiol Oncol. 2023;57(2):178–83. 37341193 10.2478/raon-2023-0022 PMC10286892

[18] Mosher V, Swain M, Pang J, Kaplan G, Sharkey K, MacQueen G, et al. Primary biliary cholangitis patients exhibit MRI changes in structure and function of interoceptive brain regions. PLoS ONE. 2019;14(2):e0211906. 30735529 10.1371/journal.pone.0211906 PMC6368379

[19] D’Mello C, Swain MG. Liver-brain inflammation axis. Am J Physiol Gastrointest Liver Physiol. 2011;301(5):G749–61. 21868631 10.1152/ajpgi.00184.2011

[20] D’Mello C, Swain MG. Liver-brain interactions in inflammatory liver diseases: implications for fatigue and mood disorders. Brain Behav Immun. 2014;35:9–20. 24140301 10.1016/j.bbi.2013.10.009

[21] D’Mello C, Swain MG. Immune-to-brain communication pathways in inflammation-associated sickness and depression. Curr Top Behav Neurosci. 2017;31:73–94. 27677781 10.1007/7854_2016_37

[22] D’Mello C, Almishri W, Liu H, Swain MG. Interactions Between Platelets and Inflammatory Monocytes Affect Sickness Behavior in Mice With Liver Inflammation. Gastroenterology. 2017;153(5):1416–e282. 28802564 10.1053/j.gastro.2017.08.011

[23] D’Mello C, Le T, Swain MG. Cerebral microglia recruit monocytes into the brain in response to tumor necrosis factoralpha signaling during peripheral organ inflammation. J Neurosci. 2009;29(7):2089–102. 19228962 10.1523/JNEUROSCI.3567-08.2009 PMC6666330

[24] D’Mello C, Riazi K, Le T, Stevens KM, Wang A, McKay DM, et al. P-selectin-mediated monocyte-cerebral endothelium adhesive interactions link peripheral organ inflammation to sickness behaviors. J Neurosci. 2013;33(37):14878–88. 24027287 10.1523/JNEUROSCI.1329-13.2013 PMC6705165

[25] D’Mello C, Ronaghan N, Zaheer R, Dicay M, Le T, MacNaughton WK, et al. Probiotics Improve Inflammation-Associated Sickness Behavior by Altering Communication between the Peripheral Immune System and the Brain. J Neurosci. 2015;35(30):10821–30. 26224864 10.1523/JNEUROSCI.0575-15.2015 PMC6605112

[26] Yao Z, van Velthoven CTJ, Kunst M, Zhang M, McMillen D, Lee C, et al. A high-resolution transcriptomic and spatial atlas of cell types in the whole mouse brain. Nature. 2023;624(7991):317–32. 38092916 10.1038/s41586-023-06812-z PMC10719114

[27] Tag CG, Weiskirchen S, Hittatiya K, Tacke F, Tolba RH, Weiskirchen R. Induction of experimental obstructive cholestasis in mice. Lab Anim. 2015;49(1 Suppl):70–80. 25835740 10.1177/0023677214567748

[28] Tag CG, Sauer-Lehnen S, Weiskirchen S, Borkham-Kamphorst E, Tolba RH, Tacke F et al. Bile duct ligation in mice: induction of inflammatory liver injury and fibrosis by obstructive cholestasis. J Vis Exp. 2015(96):52438. 10.3791/52438 PMC4354634 25741630

[29] Rodríguez-Garay EA. Cholestasis: human disease and experimental animal models. Ann Hepatol. 2003;2(4):150–8. 15115953

[30] Swain MG, Jones DEJ. Fatigue in chronic liver disease: New insights and therapeutic approaches. Liver Int. 2019;39(1):6–19. 29935104 10.1111/liv.13919

[31] Mells GF, Pells G, Newton JL, Bathgate AJ, Burroughs AK, Heneghan MA, et al. Impact of primary biliary cirrhosis on perceived quality of life: the UK-PBC national study. Hepatology. 2013;58(1):273–83. 23471852 10.1002/hep.26365

[32] Al-Harthy N, Kumagi T, Coltescu C, Hirschfield GM. The specificity of fatigue in primary biliary cirrhosis: Evaluation of a large clinic practice. Hepatology. 2010;52(2):562–70. 20683955 10.1002/hep.23683

[33] Almishri W, Altonsy MO, Swain MG. Cholestatic liver disease leads to significant adaptative changes in neural circuits regulating social behavior in mice to enhance sociability. Biochim Biophys Acta Mol Basis Dis. 2024;1870(4):167100. 38412926 10.1016/j.bbadis.2024.167100

[34] Wang Y, Guo L, Yin X, McCarthy EC, Cheng MI, Hoang AT et al. Pathogenic TNF-α drives peripheral nerve inflammation in an Aire-deficient model of autoimmunity. Proc Natl Acad Sci U S A. 2022;119(4):e2114406119 10.1073/pnas.2114406119 PMC8795502 35058362

[35] Chaudhuri A, Behan PO. Fatigue and basal ganglia. J Neurol Sci. 2000;179(1):34–42. 11054483 10.1016/s0022-510x(00)00411-1

[36] Sivakumar T, Kowdley KV. Anxiety and Depression in Patients with Primary Biliary Cholangitis: Current Insights and Impact on Quality of Life. Hepatic Medicine: Evid Res. 2021;13(null):83–92. 10.2147/HMER.S256692 PMC8409764 34483690

[37] Espinoza Lopez D, Toledo Galvan R, Jourdan Rodriguez MB, Lopez Ladron de Guevara V, Higuera de la Tijera MdF. NEUROCOGNITIVE IMPAIRMENT ASSESSMENT IN PATIENTS WITH PRIMARY BILIARY CHOLANGITIS USING THE MINI-MENTAL STATE EXAMINATION IN A TERTIARY CARE HOSPITAL. Ann Hepatol. 2025;30:102080.

[38] Wunsch E, Stadnik A, Kruk B, Szczepankiewicz B, Kotarska K, Krawczyk M, et al. Chronic Fatigue Persists in a Significant Proportion of Female Patients After Transplantation for Primary Sclerosing Cholangitis. Liver Transpl. 2021;27(7):1032. 33641247 10.1002/lt.26033

[39] Gore JC. Principles and practice of functional MRI of the human brain. J Clin Invest. 2003;112(1):4–9. 12840051 10.1172/JCI19010 PMC162295

[40] Yeo SS, Chang PH, Jang SH. The ascending reticular activating system from pontine reticular formation to the thalamus in the human brain. Front Hum Neurosci. 2013;7:416. 23898258 10.3389/fnhum.2013.00416 PMC3722571

[41] Hwang K, Shine JM, Bruss J, Tranel D, Boes A. Neuropsychological evidence of multi-domain network hubs in the human thalamus. Elife. 2021;10:e69480. 34622776 10.7554/eLife.69480 PMC8526062

[42] Seok JM, Cho W, Son D-H, Shin JH, Cho EB, Kim ST, et al. Association of subcortical structural shapes with fatigue in neuromyelitis optica spectrum disorder. Sci Rep. 2022;12(1):1579. 35091634 10.1038/s41598-022-05531-1 PMC8799731

[43] Clark AL, Sorg SF, Holiday K, Bigler ED, Bangen KJ, Evangelista ND, et al. Fatigue Is Associated With Global and Regional Thalamic Morphometry in Veterans With a History of Mild Traumatic Brain Injury. J Head Trauma Rehabil. 2018;33(6):382–92. 29385016 10.1097/HTR.0000000000000377 PMC6066453

[44] Wang J, Zhang H, Fang Y, Dong Y, Chao X, Xiao L, et al. Functional connectome hierarchy of thalamus impacts fatigue in acute stroke patients. Cereb Cortex. 2024;34(2):bhad534. 38212287 10.1093/cercor/bhad534

[45] Elvsashagen T, Shadrin A, Frei O, van der Meer D, Bahrami S, Kumar VJ, et al. The genetic architecture of the human thalamus and its overlap with ten common brain disorders. Nat Commun. 2021;12(1):2909. 34006833 10.1038/s41467-021-23175-z PMC8131358

[46] Tastan B, Heneka MT. The impact of neuroinflammation on neuronal integrity. Immunol Rev. 2024;327(1):8–32. 39470038 10.1111/imr.13419

[47] Pfisterer U, Khodosevich K. Neuronal survival in the brain: neuron type-specific mechanisms. Cell Death Dis. 2017;8(3):e2643. 28252642 10.1038/cddis.2017.64 PMC5386560

[48] Krueger DD, Tuffy LP, Papadopoulos T, Brose N. The role of neurexins and neuroligins in the formation, maturation, and function of vertebrate synapses. Curr Opin Neurobiol. 2012;22(3):412–22. 22424845 10.1016/j.conb.2012.02.012

[49] Nelson TJ, Alkon DL. Molecular regulation of synaptogenesis during associative learning and memory. Brain Res. 2015;1621:239–51. 25485772 10.1016/j.brainres.2014.11.054

[50] Gaesser JM, Fyffe-Maricich SL. Intracellular signaling pathway regulation of myelination and remyelination in the CNS. Exp Neurol. 2016;283(Pt B):501–11. 26957369 10.1016/j.expneurol.2016.03.008 PMC5010983

[51] Zhang D, Raichle ME. Disease and the brain’s dark energy. Nat Rev Neurol. 2010;6(1):15–28. 20057496 10.1038/nrneurol.2009.198

[52] Halassa MM, Sherman SM. Thalamocortical Circuit Motifs: A General Framework. Neuron. 2019;103(5):762–70. 31487527 10.1016/j.neuron.2019.06.005 PMC6886702

[53] Li Y, Wang J, Yang T, Zhang P, Ai K, Li M, et al. Alterations of Thalamic Nuclei Volumes and the Intrinsic Thalamic Structural Network in Patients with Multiple Sclerosis-Related Fatigue. Brain Sci. 2022;12(11):1538. 36421863 10.3390/brainsci12111538 PMC9688890

[54] Qian S, Wang X, Qu X, Zhang P, Li Q, Wang R, et al. Links Between the Amplitude Modulation of Low-Frequency Spontaneous Fluctuation Across Resting State Conditions and Thalamic Functional Connectivity. Front Hum Neurosci. 2019;13:199. 31263405 10.3389/fnhum.2019.00199 PMC6584839

[55] Kok A. Cognitive control, motivation and fatigue: A cognitive neuroscience perspective. Brain Cogn. 2022;160:105880. 35617813 10.1016/j.bandc.2022.105880

[56] Roy DS, Zhang Y, Halassa MM, Feng G. Thalamic subnetworks as units of function. Nat Neurosci. 2022;25(2):140–53. 35102334 10.1038/s41593-021-00996-1 PMC9400132

[57] Cunningham C. Microglia and neurodegeneration: the role of systemic inflammation. Glia. 2013;61(1):71–90. 22674585 10.1002/glia.22350

[58] Lasselin J, Lekander M, Benson S, Schedlowski M, Engler H. Sick for science: experimental endotoxemia as a translational tool to develop and test new therapies for inflammation-associated depression. Mol Psychiatry. 2021;26(8):3672–83. 32873895 10.1038/s41380-020-00869-2 PMC8550942

[59] Marsland AL, Gianaros PJ, Kuan DC, Sheu LK, Krajina K, Manuck SB. Brain morphology links systemic inflammation to cognitive function in midlife adults. Brain Behav Immun. 2015;48:195–204. 25882911 10.1016/j.bbi.2015.03.015 PMC4508197

[60] Gäbele E, Froh M, Arteel GE, Uesugi T, Hellerbrand C, Schölmerich J, et al. TNFalpha is required for cholestasis-induced liver fibrosis in the mouse. Biochem Biophys Res Commun. 2009;378(3):348–53. 18996089 10.1016/j.bbrc.2008.10.155 PMC5052129

[61] Osawa Y, Hoshi M, Yasuda I, Saibara T, Moriwaki H, Kozawa O. Tumor necrosis factor-α promotes cholestasis-induced liver fibrosis in the mouse through tissue inhibitor of metalloproteinase-1 production in hepatic stellate cells. PLoS ONE. 2013;8(6):e65251. 23755201 10.1371/journal.pone.0065251 PMC3670853

