# Supplementary material for: Thalamic homeostatic transcriptomic signatures are altered in a mouse model of cholestatic liver injury and are mitigated by systemic TNF neutralization
Source: Mol Brain. 2026 Apr 18;19:28. doi: 10.1186/s13041-026-01302-5 (PMC13097699; doi:10.1186/s13041-026-01302-5)
Supplement: Supplementary file 6 — Supplementary Material 6 [file 13041_2026_1302_MOESM6_ESM.docx]

**Supplementary information to:**

Thalamic homeostatic transcriptomic signatures are altered in a mouse model of cholestatic liver injury and are mitigated by systemic TNF neutralization.

**Table of Contents**

**Supplementary Materials and Methods**

In vivo brain Magnetic resonance imaging (MRI)…………………………………………….2

***(a)*** *In Vivo MRI Acquisition ………………………………………………………………………....2*

***(b)*** *MRI Image Analysis Workflow…………………………………………………………………...2*

RNA isolation and RT-quantitative PCR (RT-qPCR) …………………………………………2

Thalamus Transcriptome Analysis………………………………………………………….....3

i) mRNA sequencing data acquisition ………………………………………………...…….3

*ii) Transcriptome Analysis Using CLC Genomics Workbench ……………………………….…3*

*iii)* *Ingenuity Pathway analysis* *………………………………………………………………......….****4***

a) Canonical Pathways analysis …………………………………………………….……….5

b) Diseases and BioFunctions Analysis…………………………………………………..…5

c) Regulator Effects analysis……………………………………………………….……......5

**Supplemental Figures**

**Supplemental Figure 1**. Hepatic injury and cholestasis serum biomarkers in sham and bile duct ligated (BDL) mice with and without anti-TNFα Treatment …………………………………….…6

**Supplemental Figure 2**. Volcano plots illustrating differential mRNA expression for selected pairwise comparisons…………………………………………………………………………………...7

**Supplemental Figure 3**. qRT-PCR identifies thalamic gene expression signatures consistent with reduced cellular proliferation and impaired myelination after bile duct ligation …...............................8

**Supplemental Figure 4**. qPCR Analysis Shows Anti‑TNFα treatment modulates thalamic genes governing cell Growth and neural signaling………………………………………………..…9

**Supplemental Tables**

**Supplemental Table 1**. Primer sequences ………………………………….………….…….10

Supplementary References………………………………………………………………...….11

**Supplementary Materials and Methods**

**In vivo brain magnetic resonance imaging (MRI)**

***(a)*** *In Vivo MRI Acquisition*

Ten days post-BDL or sham surgery in vivo MRI imaging was conducted by personnel from the Experimental Imaging Centre (EIC) at the University of Calgary using a 9.4T/21cm horizontal bore magnet (Magnex, UK) with Bruker B-GA12S gradient insert and Bruker Avance II Biospin MR imaging system run by the ParaVision 5.1 software. Bruker’s 20mm 1H Mouse Brain Quadrature Transmit/Receive Surface CryoProbe cooled by a closed-cycle refrigeration system was used for imaging. Mice were initially anesthetized using 2.5–3% isoflurane and maintained under anesthesia with 2 % isoflurane in a gas mixture of 30% oxygen and 70% nitrogen throughout the imaging process. Temperature and respiration rate were continuously monitored during the procedure. Images were acquired using FLASH sequence (Fast Low Angle Shot) with the following parameters: TR=1500 ms, TE=6.5 ms, α=60°, FOV=19.2mm x 19.2mm, Matrix=512 x 512, 60 contiguous slices of 0.25mm giving the voxel dimensions of 0.0375 × 0.0375 × 0.25 mm. The total scan time was about 38 minutes.

***(b)*** *MRI Image Analysis Workflow*

MeVisLab software was utilized for visualization and initial preprocessing of the MRI images, including cropping to remove large, unnecessary background areas and intensity correction to standardize signal intensity across images. To accurately define brain regions and structures and compute their volumes in each mouse brain, we employed an automated image registration-based method using NiftyReg software. Our MRI data were co-registered to a high-resolution, manually segmented neuroanatomy MRI atlas of the C57Bl/6j mouse as described previously (1). All image co-registrations were further inspected visually and manually adjusted as necessary by experienced laboratory personnel to ensure precise anatomical alignment. Following this validation, the volumes of brain structures for the left and right hemispheres were automatically calculated by NiftyReg. Subsequently, the volume of each brain structure was normalized relative to total brain volume. Finally, pairwise comparisons were conducted of these normalized brain region volumes (% of total brain volume) between BDL and sham groups using two-sided *t*-tests (2).

**RNA isolation and RT-quantitative PCR (qRT-PCR)**

At 10 days post BDL or sham surgery mice were euthanized with isoflurane and perfused with 20 ml of ice-cold PBS. The whole brain was removed, the thalamus was dissected and stored at -80°C in RA1 lysis buffer (Cat No. 740961.500; Macherey–Nagel, Düren, Germany) until RNA extraction. Distinct cohorts of mice were used for the MRI study and RT-qPCR/RNA-Seq analyses. Total RNA was extracted using the NucleoSpin® RNA purification kit (Cat No. 740955-250, Macherey–Nagel, Düren, Germany). The synthesis of complementary DNA (cDNA) was then performed using the qScript cDNA Synthesis kit (Cat No. CA101414-098, Quanta Biosciences, Massachusetts, USA). For reverse transcription polymerase chain reaction (RT-PCR), we employed the PowerUp SYBR Green Master Mix (Cat No. A25742, Life Technologies, California, USA) in conjunction with a QuantStudio 3 Real-Time PCR System (Applied Biosystems, Foster City, CA). The PCR protocol included the following parameters: UDG activation at 50°C for 2 min, polymerase activation at 95°C for 2 min, denaturation at 95°C for 1 sec, and annealing/extension at 60°C for 30 sec (40 Cycles). For melt curve stage 1.6°C/sec at 95°C for 15 sec, 1.6°C/sec at 60°C for 1 min, and 0.15°C/sec at 95°C for 15 sec. The ΔΔCt (delta-delta Ct) method was used to analyze relative changes in gene expression in thalamus RNA samples. Differences in input cDNA were corrected for by normalizing to a reference housekeeping gene, Gapdh. This was followed by calculating ΔΔCt values to convert the raw Ct values of gene expression into a relative expression fold change between the experimental group and the calibrator group. All oligonucleotides were obtained from Integrated DNA Technologies (IDT, Coralville, IA). Customized primers were designed using IDT's software and were validated for specificity through Primer-BLAST and melt-curve analyses (3). Primer sequences used for qPCR amplification are provided in Supplemental Table 1.

**Thalamus Transcriptome Analysis**

i) mRNA sequencing data acquisition

Bulk tissue RNA sequencing on extracted RNA from the thalamus was conducted by the University of Calgary Centre for Health Genomics and Informatics. The RNA Integrity Number (RIN) was determined for each RNA sample. Samples with a RIN score higher than eight were considered good quality. Poly-A mRNA-seq libraries from such samples were prepared using the Ultra II Directional RNA Library kit (New England BioLabs Inc., MA, US) according to the manufacturer’s instructions. Libraries were then quantified using the Kapa qPCR Library Quantitation kit (Roche Sequencing Solutions, Pleasanton, CA, USA) according to manufacturer’s directions (3). Ultimately, RNA libraries were sequenced using paired-end 50 bp fragment sequencing on NovaSeq™ 6000 and NextSeq 2000 high-throughput Illumina sequencing systems for the first and second cohorts, respectively. A minimum of 32 million read pairs per sample was achieved in both RNA-seq runs. A total of nine thalamic samples per treatment group were sequenced and subsequently combined for bulk RNA-seq analysis, except for the BDL + anti-TNFα group, for which four thalamic samples from the second cohort were analyzed.

*ii) Transcriptome Analysis Using CLC Genomics Workbench*

We utilized CLC Genomics Workbench version 24.0.2 to perform bulk RNA-seq analysis. Raw FASTQ files containing sequencing reads generated from the Illumina platform were imported into CLC using the Illumina High-Throughput Sequencing Import tool. Paired-end reads were imported with the read orientation set as "Forward Reverse." Default parameters were maintained, including a minimum distance of 1 and a maximum distance of 1,000 and the option "remove failed reads" was enabled. The resulting combined paired-end sequencing reads were subsequently trimmed before assembly and mapping using the CLC Trim Reads 3.0 tool. The following trimming parameters were enabled to ensure high-quality data: Quality Score-based Trimming, Ambiguous Nucleotide Removal, Automatic Adapter Trimming, and the options Remove on First Read and Remove on Second Read. Additional parameter settings included a quality limit of 0.05, a maximum of 2 ambiguous nucleotides per read, a maximum read length of 150 nucleotides, and trimming specifically from the 3'-end.

The CLC RNA-Seq Analysis 2.8 tool was used to map RNA-Seq reads to a reference transcriptome by aligning sequences to known genes and quantifying gene expression levels based on the number of reads mapped to each gene. This analysis was performed using the following parameters: the reference type was set as a genome annotated with genes and transcripts based on the Mus musculus (GRCm39) reference sequence. Gene annotation was carried out using the Ensembl Mus musculus gene track (GRCm39.112), along with the corresponding mRNA track (GRCm39.112_mRNA). Alignment parameters included a mismatch cost of 2, an insertion cost of 3, a deletion cost of 3, a length fraction of 0.8, and a similarity fraction of 0.8. The analysis was performed with reverse strand specificity, allowing a maximum of 10 hits per read. Broken read pairs were ignored, and expression levels were reported as total counts (4). In addition to total counts, CLC Genomics Workbench offers various formats for RNA-seq read quantification and normalization. These include TPM (Transcripts Per Million), FPKM (Fragments Per Kilobase Million), and TMM-normalized counts (Trimmed Mean of M-values), which we used in downstream differential expression analysis.

The Gene Expression Tracks (GE) generated in the previous step were used to perform statistical differential expression analysis using the **CLC Differential Expression for RNA-Seq** tool version 2.9 to assess gene expression differences based on treatment type. The Differential Expression for RNA-Seq tool employs the Trimmed Mean of M-values (TMM) normalization method to address composition bias and technical variability across samples. TMM normalization adjusts for differences in library sizes (total read counts per sample) and RNA population composition, facilitating accurate cross-sample comparisons (5). This tool applies multi-factorial statistics based on a negative binomial generalized linear model (GLM) and allows for control of batch effects (6). In this analysis, we examined gene expression differences based on treatment type while accounting for batch effects as a potential confounding factor since the RNA-Seq data were derived from two separate experiments. Additional parameters included enabling the option to “downweight outliers” and applying a filter based on average gene expression for false discovery rate (FDR) correction.

***iii) Ingenuity Pathway Analysis***

Statistical comparison tracks generated by CLC were exported to the web-based Ingenuity Pathway Analysis (IPA) software (IPA; QIAGEN, Redwood City, Version 134816949) to uncover biological insights underlying the observed differences in tissue gene expression. Differentially expressed genes (DEGs) in the thalamus meeting the predefined cutoff criteria, including a false discovery rate (FDR) of ≤0.05, an absolute fold change of ≥1.2, and a minimum group mean expression of ≥1 FPKM (Fragments Per Kilobase of transcript per Million mapped reads), were classified as analysis-ready molecules. These analysis-ready molecules were then used in IPA’s Core Analysis to identify relevant pathways, networks, and functional associations.

In the Core Analysis, we used Ingenuity Knowledge Base (Genes Only) as the reference set, and the "relationships to consider" option was set to include both direct and indirect relationships for assembling networks and identifying transcriptional regulators (7-9). Additionally, the option to score master regulators for relationships to diseases, functions, genes, or chemicals was employed using the "score with causal paths only" option (10). For the Node Types Filter, which restricts the types of molecules included in the analysis, groups, and complexes were excluded from the Core Analysis based on recommendations from Qiagen experts (11). All accessible data sources on the IPA server were utilized and experimentally observed confidence levels for pathway construction were used and considered exclusively. Additionally, we imposed no restrictions on species, tissue, or mutation types while constructing the interaction networks (3).

*a) Canonical Pathways analysis*

We used the pathway analysis tool within IPA software to identify canonical signaling pathways in the thalamus impacted by cholestatic liver disease. A canonical pathway refers to a well-established series of molecular events that occur in cells, representing the most prevalent pathway for a specific biological process (12). For each canonical pathway, IPA computed Fisher's exact test p-value to assess the statistical significance of differentially expressed gene (DEG) enrichment in the pathway, compared to what might be expected by chance from the total number of unique genes in the input list. These p-values were adjusted for multiple testing using the Benjamini and Hochberg False Discovery Rate (FDR) method. We deemed a canonical pathway to be significantly enriched (impacted) if its FDR-adjusted p-value is p≤ 0.05. Furthermore, the software can predict the activation (positive Z-score), or inhibition (negative Z-score) of canonical pathways, upstream regulators, or downstream functions based on the directional measurements of DEGs provided to IPA. When directionality is ambiguous, a null value is displayed. An absolute z-score ≥2 is considered significant for activation prediction for enriched pathways.

*b) Diseases and BioFunctions Analysis:*

The Diseases and BioFunctions Analysis module in IPA is a powerful tool that enables robust interpretation of transcriptomic data by mapping gene expression changes to established disease pathways and biological functions. In our study, we utilized this tool to elucidate the impact of cholestatic liver injury on diseases and biological functions, specifically focusing on cellular growth and proliferation as well as nervous system development and function in the thalamus. For our analysis, we applied an enrichment cutoff of an FDR-adjusted p-value of 0.05 or less and an absolute predicted activation Z-score of 2 or greater. These criteria allowed systematic identification of impacted diseases and functions, along with their directionality of regulation (activated or suppressed), within their respective IPA-defined categories (13, 14).

*c) Regulator Effects analysis:*

The Regulator Effects analysis in IPA is another tool that merges upstream regulator networks with downstream effects networks to form directionally linked hypotheses, helping to predict how activated or inhibited upstream regulators might influence downstream biological functions or diseases through differentially expressed molecules in our transcriptomic dataset which connect regulators to diseases or functions (15). We implemented Regulator Effects Analysis in IPA to connect and merge upstream regulators with disease and functional outcomes, using differentially expressed genes (DEGs) from our transcriptomic thalamus data as intermediaries. This approach can reveal distinct biological networks that may cause increases or decreases in downstream phenotypic or functional outcomes that in turn are related to the control of thalamic function and size, thereby providing a potential mechanistic explanation for structural and/or functional alterations observed in the thalamus of cholestatic mice. The module produces detailed Regulator Effects networks, specifying distinct regulators, their target molecules within our dataset, and the impacted diseases/functions, along with their predicted activation or inhibition statuses. In addition, the generated networks can also be visualized in three tiers: the top tier represents the upstream regulators, the middle tier comprises our thalamic DEG dataset molecules that connect these regulators to the diseases and functions, and the bottom tier consists of the diseases, functions, or phenotypes affected by the regulators (15).

|  |
| --- |

**Suppl. Fig. 1.** Hepatic injury and cholestasis serum biomarkers in sham and bile duct ligated (BDL) mice with and without anti-TNFα Treatment.

Panels **A** and **B** depict liver damage serum biomarkers alanine aminotransferase (ALT), and aspartate aminotransferase (AST) levels measured at day 10 post bile duct ligation (BDL) or sham surgeries. Both ALT and AST levels were significantly elevated in BDL mice compared to sham mice, indicating induction of hepatocyte damage that was not impacted by anti-TNF treatment. Panels **C** and **D** depict serum total bilirubin (TBil), and alkaline phosphatase (ALP) levels measured at day 10 post-BDL or sham ligation. BDL was associated with a significant increase in levels of ALP and TBil, consistent with establishment of cholestasis. Additionally, anti-TNF did not alter ALP and TBil serum levels in BDL mice (panels C and D), indicating that cholestatic liver damage remained unaltered. For statistical annotations, ***** indicates p < 0.0001 for all BDL groups compared with sham; **^+^** indicates p < 0.0002 for BDL versus sham; **^++^** indicates p < 0.0001 for BDL + anti‑TNFα versus sham; ^$$^ indicates p < 0.0002 for BDL versus sham; ^$^ indicates p < 0.0072 for BDL + anti‑TNFα versus sham; and **^#^** indicates p < 0.0001 for all BDL groups versus sham. Each group includes 7–9 mice. For all BDL versus BDL + anti-TNF comparisons, statistical p-values were not significant and were p < 0.3045, 0.0748, 0.9175, 0.9999 for ALT, AST, ALP, and Tbil, respectively.

| **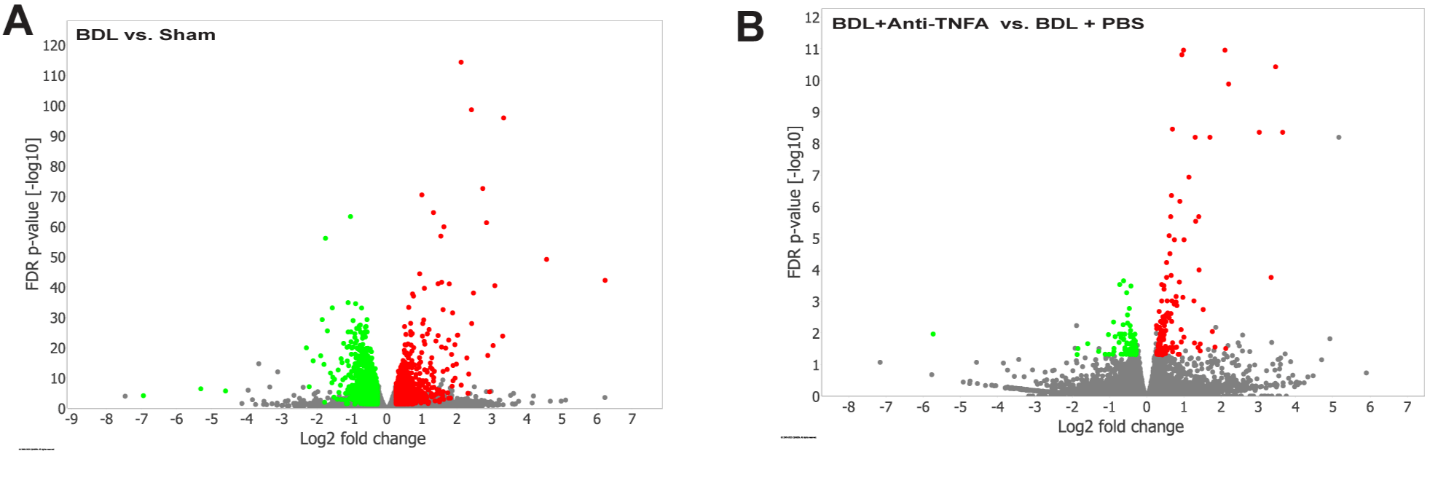** |
| --- |

**Suppl. Fig. 2**. Volcano plots illustrating differential mRNA expression for selected pairwise comparisons. (A) Volcano plot depicting differential mRNA expression between the BDL and sham groups in the thalamus. (B) Volcano plot depicting differential mRNA expression between the BDL + anti-TNFα and BDL groups in the thalamus. The X-axis represents the Log₂ fold-change, whereas the Y-axis shows the −log₁₀ adjusted p-value. Significantly differentially expressed mRNAs are highlighted: red dots represent significantly upregulated transcripts, and green dots indicate significantly downregulated transcripts. Gray dots represent mRNAs that did not meet the significance thresholds: adjusted p-value (FDR) < 0.05, absolute fold-change ≥1.2, and minimum mean expression level of 1 transcript per million (TPM).

|  |
| --- |

|  |
| --- |

**Suppl. Fig 3.** qRT-PCR identifies thalamic gene expression signatures consistent with reduced cellular proliferation and impaired myelination after bile duct ligation. Mice underwent bile duct ligation (BDL) or sham surgery and were euthanized 10 days later. Thalamic tissue was then harvested for quantitative PCR analysis. Compared with sham controls, BDL mice showed a significant reduction in the cellular proliferation marker Ki67 **(A),** an increase in the cell‑cycle inhibitor Cdkn1a **(B),** and decreased myelination‑related gene proteolipid protein 1 (Plp1) expression (encodes a key component of myelin) (**C**). Symbols *, **, ***, represent P-values: ≤ 0.0002, 0.0004, 0.0006, respectively. n = 7/8 mice per group. Genes in the figure are represented by standard gene symbols, and the corresponding official full names of these genes can be found in **Suppl. File 5.**

|  |
| --- |

**Suppl. Fig4.** qPCR Analysis shows that anti-TNF treatment modulates expression of thalamic genes governing cell proliferation and myelination in BDL mice. Mice received intraperitoneal injections of anti‑TNF antibodies every other day, starting 2 days after bile‑duct ligation, and thalamic tissue was collected on day 10 post‑surgery. Quantitative PCR showed that anti‑TNF treatment significantly elevated Ki‑67 mRNA levels in the thalamus consistent with increased cellular proliferation **(A).** Anti-TNF treatment did not significantly alter thalamic expression of Plp1 or Cdkn1a mRNA levels **(B and C).** *P < 0.019; n = 6–8 mice per group. For Cdkn1a and Plp1p-values were not significant and were p < 0.5934, 0.1008 respectively. Genes in the figure are represented by standard gene symbols, and the corresponding official full names of these genes can be found in **Suppl. File 5.**

|  |
| --- |

| **Gene name** | **Q-PCR Primers’ sequence (5' to 3')** |
| --- | --- |
| mki67 | Forward: AACCATCATTGACCGCTCCTTT |
|  | Reverse: ATCTTGACCTTCCCCATCAGGG |
| Cdkn1a | Forward: TCCAGACATTCAGAGCCACAGG |
|  | Reverse: GTCAAAGTTCCACCGTTCTCGG |
| Plp1 | Forward: CCTATGCCCTGACTGTTGTATG |
|  | Reverse: GTCTTGCTAGGGAAGGCAATAG |
| Ppp1r1b | Forward: GCGAAGAGGTTAAAGCCAGA |
|  | Reverse: CCAGAACTCGGCGTTGTATTA |
| Kcnh3 | Forward: GGCAAGCACAAGCTCAATAAG |
|  | Reverse: CCACAGTGCAGCAGGATAAA |
| Kcnc4 | Forward: GGTGGTCTGTCTTCATCGTTAG |
|  | Reverse: CCATACACGTGGTCAGAAGAAA |
| Gpr88 | Forward: CAAGCCACACGTTAGGAAGTA |
|  | Reverse: CTGTCTGCCTGATTCCATCTT |
| Egr4 | Forward: AGGCACTTCCTTGGGACTGAAG |
|  | Reverse: GGTACATCCCCAGCTTGTCTCT |
| Adcy1 | Forward: CTGTCTTCTCTGTGGTGTCTTG |
|  | Reverse: AGAGCACTCGTGGGTATAGT |

**Supplementary Table 1**. Primer sequences used for real-time quantitative reverse transcriptase polymerase chain reaction (qRT-PCR).

**Supplementary References**

1. Dorr AE, Lerch JP, Spring S, Kabani N, Henkelman RM. High resolution three-dimensional brain atlas using an average magnetic resonance image of 40 adult C57Bl/6J mice. Neuroimage. 2008;42(1):60-9.

2. Nadkarni R, Han Z, Anderson R, Allphin A, Clark D, Badea A, et al. Volumetric brain region segmentation and morphometry in mouse models using high-resolution hybrid micro-CT imaging: SPIE; 2024.

3. Almishri W, Altonsy MO, Swain MG. Cholestatic liver disease leads to significant adaptative changes in neural circuits regulating social behavior in mice to enhance sociability. Biochim Biophys Acta Mol Basis Dis. 2024;1870(4):167100.

4. Wagner GP, Kin K, Lynch VJ. Measurement of mRNA abundance using RNA-seq data: RPKM measure is inconsistent among samples. Theory in Biosciences. 2012;131(4):281-5.

5. Liu C-H, Di YP. Analysis of RNA Sequencing Data Using CLC Genomics Workbench. In: Keohavong P, Singh KP, Gao W, editors. Molecular Toxicology Protocols. New York, NY: Springer US; 2020. p. 61-113.

6. Pai CS, Huang JT, Lu X, Simons DM, Park C, Chang A, et al. Clonal Deletion of Tumor-Specific T Cells by Interferon-γ Confers Therapeutic Resistance to Combination Immune Checkpoint Blockade. Immunity. 2019;50(2):477-92.e8.

7. Zagare A, Balaur I, Rougny A, Saraiva C, Gobin M, Monzel AS, et al. Deciphering shared molecular dysregulation across Parkinson’s disease variants using a multi-modal network-based data integration and analysis. npj Parkinson's Disease. 2025;11(1):63.

8. Jung SH, Brownlow ML, Pellegrini M, Jankord R. Divergence in Morris Water Maze-Based Cognitive Performance under Chronic Stress Is Associated with the Hippocampal Whole Transcriptomic Modification in Mice. Front Mol Neurosci. 2017;10:275.

9. Qu JH, Tarasov KV, Tarasova YS, Chakir K, Lakatta EG. Transcriptome of Left Ventricle and Sinoatrial Node in Young and Old C57 Mice. Fortune J Health Sci. 2023;6(3):332-56.

10. Krämer A, Green J, Pollard J, Jr., Tugendreich S. Causal analysis approaches in Ingenuity Pathway Analysis. Bioinformatics. 2014;30(4):523-30.

11. QIAGEN. IPA Spring Release (2016): Release Notes. Redwood City, CA; 2016.

12. Ackers I, Malgor R. Interrelationship of canonical and non-canonical Wnt signalling pathways in chronic metabolic diseases. Diab Vasc Dis Res. 2018;15(1):3-13.

13. Lyu M, Yan C-L, Liu H-X, Wang T-Y, Shi X-H, Liu J-P, et al. Network pharmacology exploration reveals endothelial inflammation as a common mechanism for stroke and coronary artery disease treatment of Danhong injection. Scientific Reports. 2017;7(1):15427.

14. Amit M, Xie T, Gleber-Netto FO, Hunt PJ, Mehta GU, Bell D, et al. Distinct immune signature predicts progression of vestibular schwannoma and unveils a possible viral etiology. J Exp Clin Cancer Res. 2022;41(1):292.

15. QIAGEN. Regulator Effects in IPA. 2023.
